# Urban–rural and geographic differences in overweight and obesity in four sub-Saharan African adult populations: a multi-country cross-sectional study

**DOI:** 10.1186/s12889-016-3789-z

**Published:** 2016-10-28

**Authors:** IkeOluwapo O. Ajayi, Clement Adebamowo, Hans-Olov Adami, Shona Dalal, Megan B. Diamond, Francis Bajunirwe, David Guwatudde, Marina Njelekela, Joan Nankya-Mutyoba, Faraja S. Chiwanga, Jimmy Volmink, Robert Kalyesubula, Carien Laurence, Todd G. Reid, Douglas Dockery, David Hemenway, Donna Spiegelman, Michelle D. Holmes

**Affiliations:** 1 Department of Epidemiology and Medical Statistics, Faculty of Public Health, College of Medicine, University of Ibadan, Ibadan, Nigeria; 2 Institute of Human Virology, Abuja, Nigeria; 3 School of Medicine Greenbaum Cancer Center and Institute of Human Virology, University of Maryland, Baltimore, MD USA; 4 Department of Medical Epidemiology and Biostatistics, Karolinska Institute, Stockholm, Sweden; 5 Department of Epidemiology, Harvard T. H. Chan School of Public Health, Boston, MA USA; 6 Department of Community Health, Mbarara University of Science and Technology, Mbarara, Uganda; 7 Department of Epidemiology & Biostatistics, Makerere School of Public Health, Kampala, Uganda; 8 Department of Physiology, Muhimbili University of Health and Allied Sciences, Dar es Salaam, Tanzania; 9 Department of Internal Medicine, Muhimbili National Hospital, Dar es Salaam, Tanzania; 10 The South African Cochrane Centre, South African Medical Research Council, Cape Town, South Africa; 11 Centre for Evidence-based Health Care, Faculty of Medicine and Health Sciences, Stellenbosch University, Cape Town, South Africa; 12 Department of Environmental Health, Harvard T. H. Chan School of Public Health, Boston, MA USA; 13 Department of Health Policy and Management, Harvard T. H. Chan School of Public Health, Boston, MA USA; 14 Channing Division of Network Medicine, Department of Medicine, Brigham and Women’s Hospital and Harvard Medical School, Boston, MA USA

**Keywords:** Prevalence of obesity and overweight, risk factors for over-nutrition, Sub-Saharan Africa, South Africa, Nigeria, Tanzania, Uganda

## Abstract

**Background:**

Overweight and obesity are on the rise in developing countries including sub-Saharan Africa. We undertook a four-country survey to show the collective burden of these health conditions as they occur currently in sub-Saharan Africa and to determine the differences between urban and rural populations and other socio-economic factors.

**Methods:**

Participants were nurses in two hospitals in Nigeria (200), school teachers in South Africa (489) and Tanzania (229), and village residents in one peri-urban (297) and one rural location in Uganda (200) who completed a standardised questionnaire. Their height and weight were measured and body mass index calculated. Factor analysis procedure (Principal component) was used to generate a wealth index. Univariate and multivariate analyses with binary logistic regression models were conducted to examine the associations between potential correlates and the prevalence of overweight and obesity with 95 % confidence intervals.

**Results:**

The prevalence of overweight and obese (combined) was 46 %, 48 %, 68 %, 75 % and 85 % in rural Uganda, peri-urban Uganda, Nigeria, Tanzania and South Africa (SA), respectively. Rural Uganda, Peri- urban Uganda, Nigeria, Tanzania and SA had obesity prevalence of 10 %, 14 %, 31 %, 40 % and 54 %, respectively (*p* < 0.001). Overall, prevalence of overweight was 374 (31 %) and obesity, 414 (34 %). Female sex was a predictor of overweight and obesity (combined) in peri-urban Uganda [AOR = 8.01; 95 % CI: 4.02, 15.96) and obesity in rural Uganda [AOR = 11.22; 95%CI: 2.27, 55.40), peri-urban Uganda [AOR = 27.80; 95 % CI: 7.13, 108.41) and SA [AOR = 2.17; 95 % CI: 1.19, 4.00). Increasing age was a predictor of BMI > =25 kg/m^2^ in Nigeria [Age > =45 - AOR = 9.11; 95 % CI: 1.72, 48.16] and SA [AOR = 6.22; 95 % CI: 2.75, 14.07], while marital status was predictor of BMI > =25 kg/m^2^ only in peri-urban Uganda. [Married - AOR = 4.49; 95 % CI: 1.74, 11.57]. Those in Nigeria [AOR = 2.56; 95 % CI: 1.45, 4.53], SA [AOR = 4.97; 95 % CI: 3.18, 7.78], and Tanzania [AOR = 2.68; 95 % CI: 1.60, 4.49] were more likely to have BMI > =25 kg/m^2^ compared with the rural and peri-urban sites.

**Conclusion:**

The high prevalence of overweight and obesity in these sub-Saharan African countries and the differentials in prevalence and risk factors further highlights the need for urgent focused intervention to stem this trend, especially among women, professionals and urban dwellers.

## Background

Non-communicable diseases (NCDs) such as cardiovascular diseases (CVDs), type 2 diabetes, musculoskeletal disorders and cancers have been reported as the major causes of death globally and they accounted for 36 million (63 %) of the 57 million deaths in 2008 [1]. Nearly 80 % of these NCD deaths, equivalent to 29 million people, occurred in low and middle income countries with the projection of about 52 million deaths annually by 2030 [1]. This high rate is attributed to the "epidemiologic transition" from communicable to non-communicable diseases [2, 3]. Increasing prevalence of NCD risk factors such as physical inactivity, unhealthy diet, alcohol consumption, and cigarette smoking have been reported among populations in low and middle-income countries (LMICs) and attributed to urbanization, industrialization, globalization and lifestyle changes [4]. One major consequence of these changes is a nutrition transition, the harbinger of overweight and obesity which are important modifiable risk factors for chronic NCDs [5, 6]. The nutrition transition results in distortion and extinction of indigenous and traditional food habits which are healthier than the westernized habit of energy dense food consumption [7].

Overweight and obesity have become major global health challenges. In 2010, overweight and obesity were estimated to cause 3 · 4 million deaths, 3 · 9 % of years of life lost, and 3 · 8 % of disability-adjusted life-years (DALYs) worldwide [1]. According to the World Health Organisation (WHO), in 2014, more than 1.9 billion adults (39 %), 18 years and older, were overweight. Of these, over 600 million (13 %) were obese (Body Mass Index- BMI ≥ 30.0 Kg/m^2^) [8]. Overall in Africa, currently some 27 % of adults aged 20 years and over are overweight, and 8 % are obese [9]. The WHO estimates that overweight and obesity have increased drastically in sub-Saharan African (SSA) [10]. Among sub-Saharan African men in 2013, Equatorial Guinea had the highest prevalence of obesity (25 %) and Uganda the least (1.7 %) whereas among women, South Africa had the highest (42 %) and Ethiopia the least prevalence (1.8 %) [11].

The prevalence of overweight and obesity and its temporal trends vary in magnitude by numerous factors including sex, age, socio-economic status, diet, physical activity and geographic location [12]. Urbanization, which comes with increased access to energy-dense foods and less strenuous jobs is a risk factor [13]. It is currently estimated that as much as 20–50 % of urban populations in Africa are classified as either overweight or obese [13].

Due to the unique genetic diversity and enormous heterogeneity in life-styles in different countries, the burden of obesity and overweight and their determinants need to be studied in different sub-Saharan populations. Up-to-date information about levels and trends in overweight and obesity is essential both to quantify the resultant health effects of nutrition transition and to prompt decision makers to prioritize action and assess progress.

To this end, we undertook a series of pilot studies as preparation for a multi-country large-scale longitudinal study in sub-Saharan Africa named the Africa/Harvard T. Chan School of Public Health (HSPH) Partnership for Cohort Research and Training (PaCT). This study was carried out in four sub-Saharan countries at five different sites (Nigeria, South Africa, Tanzania, and one peri-urban and one rural site in Uganda). In this report we present a subset of the findings highlighting the patterns of overweight and obesity at these sites, the prevalence in relation to other socio-economic factors and differences between urban and rural populations.

## Methods

The PaCT study sites and participants have been described in detail elsewhere [14]. Briefly, these 1463 participants included nurses in two hospitals in Nigeria, school teachers in South Africa and Tanzania, and village residents in one peri-urban and one rural location in Uganda. All sites used random selection for participants. Participants were adults aged 18 years or older.

A standardized questionnaire was used at all sites. Some questions were adapted from the World Health Organization STEPS instrument developed for use in resource-limited countries [15]. We focused here on the sections related to overweight, obesity and their risk factors. These included questions on “ever smoking” and “number of cigarettes” in the preceding 24 h. Those with secondary level education or less were categorized as low education while those with university education or above were categorised as having high education.

Height and weight were measured after enrolment by trained nurses or study staff following standardized procedures. We calculated body mass index (BMI) as weight in kilograms divided by height in meters squared, and used the standard definitions of underweight (below 18.5 Kg/m^2^), normal weight (18.5–24.9 Kg/m^2^), overweight (25–29.9 Kg/m^2^) and obese (30 Kg/m^2^and above) [16]. Informed consent was obtained from each subject either by voluntarily posting back a signed form with a completed questionnaire (South Africa and Tanzania), or through documentation with trained interviewers (Nigeria and Uganda) [14].

### Data analysis

All data analyses were conducted with STAT version 12.0 [17]. We first summarized variables using descriptive statistics such as means, standard deviation, median and range for continuous variables and proportions for categorical variables. We then performed univariate and multivariate analyses using binary logistic regression to examine the associations between potential correlates (age, sex, education, marital status, site, smoking status, wealth index) and the BMI status. Odds Ratio and 95 % confidence intervals were presented. In logistic regression anaylses, variables with *p* < 0.5 were included in the multivariate model except variables exhibiting multicollinearity and with no representation in any of the sites such as education, occupation and wealth which were excluded; while model fit was assessed using Chi square goodness of fit test [18]. Twenty four persons who were underweight (body mass index < 18.5 kg/m2.) were excluded from the bivariate and multivariate analysis because the objective was to determine correlates of overweight and obesity comparing with normal [19]. We used factor analysis (Principal components) procedure with varimax rotation as reported by Filmer and Pritchett [20] to generate a wealth index, using the household source of drinking water and type of fuel used for cooking. The created wealth quintiles were categorised into three groups: high, being > =75th percentile, middle, from the median to < 75th percentile and low, being less than the median. Level of significance was set at 5 %.

## Results

### Respondents’ characteristics

We enrolled 1463 participants comprising 489 teachers in South Africa (33 %), 276 teachers in Tanzania (19 %), 200 nurses in Nigeria (14 %), 298 community members in peri-urban Uganda (21 %) and 200 in rural Uganda (14 %). The response rates were between 96 % and 99 % across the sites. Out of the 498 in the Uganda sites, two thirds (322, 65 %) of the participants were self-employed, 123 (25.0 %) were unemployed while 49 (9.9 %) were either in government or private employment. All the respondents in Nigeria were employed as nurses and those in South Africa and Tanzania as teachers.

The frequency distribution of the respondents’ characteristics by country site is shown in Table 1. The results for each parameter are presented based on the number that responded. Overall, two thirds were female (927, 65 %), 976 (70 %) were currently married/living together and 856 (66.0 %) were classified to have high education. Only 134 (10 %) of the participants mentioned they ever smoked a cigarette. A quarter (351, 25 %) of the participants were in the low socio-economic group while 522 (37 %) were in the high socio-economic group.

**Table 1:** Respondents’ characteristics and body mass index status by country

Characteristics	Rural Uganda	Peri-Urban Uganda	Tanzania	Nigeria	South Africa	Total
	*n* (%)	*n* (%)	*n* (%)	*n* (%)	*n* (%)	*n* (%)
Sex						
Female	100 (50)	158 (53)	192 (83)	133 (67)	344 (70)	927 (65)
Male	100 (50)	139 (47)	35 (17)	67 (34)	145 (30)	489 (35)
Age, years						
18–34	88 (46)	141 (58)	57 (27)	73 (45)	43 (9)	402 (31)
35–44	62 (33)	47 (19)	75 (36)	55 (33)	167 (34)	406 (31)
> = 45	41(21)	55 (23)	77 (37)	36(22)	276(57)	485 (38)
Range	19–65	18–80	25–60	23–57	22–71	18–80
Mean (SD) Age	37 (11)	36 (14)	42 (9)	38 (8)	46 (9)	41 (11)
Education						
Low education	198 (99)	201 (72)	0	0	0	399 (31)
High education	2 (1)	77 (28)	223 (100)	161(100)	429 (100)	892 (69)
Marital status						
Never married	9 (5)	72 (24)	27 (12)	60 (32)	75 (16)	243 (18)
Married/living together	173 (87)	179 (61)	174 (77)	121 (64)	329 (69)	976 (70)
Separated, divorced, widowed, other	18 (9)	44 (15)	26 (11)	7 (4)	71 (15)	166 (12)
Cigarette smoking						
Ever smoked	25 (13)	19 (6)	3 (1)	6 (3)	85 (19)	138 (10)
Never smoked	175 (87)	277 (94)	225 (99)	188 (97)	370 (81)	1235 (90)
Number of Cigarette in past 24 h						
<= 19	22 (88)	19 (100)	3 (100)	6 (100)	65(76)	115 (84)
> = 20	3 (12)	0	0	0	20 (24)	23 (16)
Wealth status						
High	0	5 (2)	0	77 (39)	440 (90)	522 (37)
Middle	56 (28)	180 (61)	150 (66)	107 (54)	49 (10)	542 (38)
Low	144 (72)	112 (38)	79 (35)	16 (8)	0	351 (25)
Body mass index (Kg/M^2^)						
Underweight (BMI < 18.5 kg/m^2^)	4 (2)	17 (6)	1 (<1)	1 (<1)	1 (<1)	24 (2)
Normal (BMI 18.5–24.9 Kg/m^2^)	86 (52)	161 (56)	42 (25)	37 (32)	70 (15)	396 (33)
Overweight (BMI 25–29.9 Kg/m^2^)	59 (36)	69 (24)	58 (35)	43 (37)	145 (31)	374 (31)
Obese (BMI ≥30 Kg/m^2^)	16 (10)	40 (14)	67 (40)	36 (31)	255 (54)	414 (34)
Range (BMI)	15–43	17–42	17–45	18–40	18–66	15–66
Mean (SD) BMI	25 (4)	25 (5)	29 (5)	28 (5.2)	32 (7)	28 (7)

The mean age of the participants was 41.0 ± 11.1 years and ranged from 18– 80 years. South African and Tanzanian participants were older than those from the other sites. (Analysis of variance -ANOVA (Bonferroni): F = 63.9 *p* < 0.0001).

### Prevalence of overweight and obesity

Overall, the mean body mass index was 28.4 (SD = 6.6 Kg/m^2^). The prevalence of overweight was 374 (31 %) and of obesity was 414 (34 %) (Table 1). South Africa had a statistically significant higher prevalence of obesity (54 %) compared to the other sites (*p* < 0.0001). Also Tanzanian prevalence of obesity (at 40 %) was statistically significantly higher than the two Ugandan sites but not Nigeria. (F = 80.2; *p* < 0.0001, Bonferroni (Dunn))

**Fig. 1:**
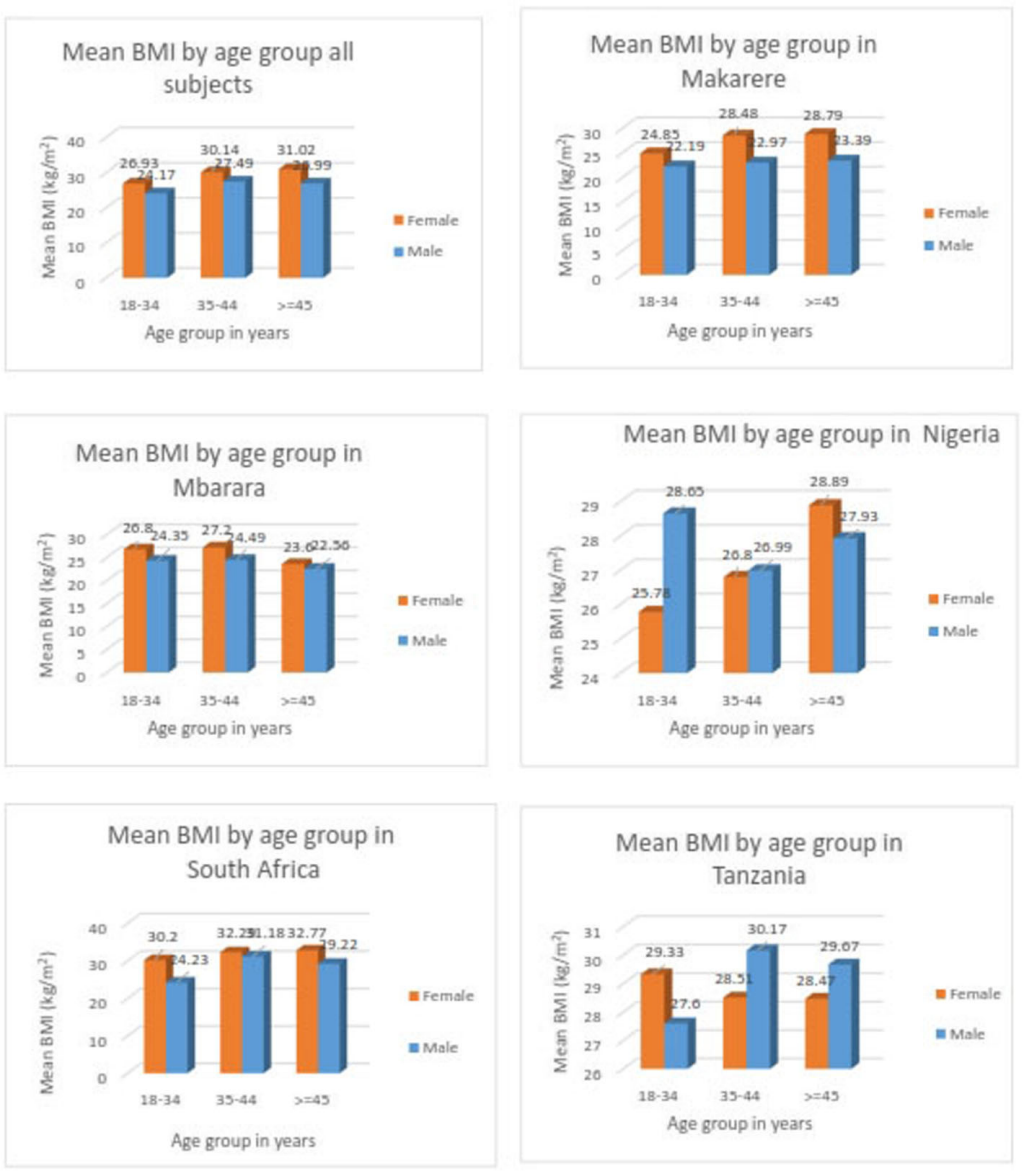
Mean BMI by age group and sex overall and in the different study sites

The association between BMI and age and site and sex is depicted in Fig. 1. This showed mean BMI to be higher among females except for respondents from Nigeria and Tanzania. In addition, the mean BMIs were significantly higher in females than males for all those above 24 years old from peri-urban Uganda and South Africa

The relationship between BMI status (underweight, normal overweight, and obese) by sex and site is shown on Table 2. Generally, women had a higher proportion of those obese except for Tanzania and Nigeria that had a higher proportion of obese men. Among South African women, 60 % were obese, while 33 % of the men were obese.

**Table 2:** BMI status by sex and site in population samples from Uganda, Tanzania, Nigeria and South Africa

	BMI Status (Kg/M^2^)				
	Underweight (BMI < 18.5 kg/m^2^)	Normal (BMI 18.5–24.9 Kg/m^2^)	Overweight (BMI 25–29.9 Kg/m^2^)	Obese (BMI ≥30 Kg/m^2^)	Total
Country site and Sex	*n* (row %)	*n* (row %)	*n* (row %)	*n* (row %)	*n* (column %)
Rural Uganda					
Male	3 (3.7)	48 (59.3)	28 (35.5)	2 (2.4)	81 (52.3)
Female	1 (1.4)	38 (51.4)	31 (41.9)	14 (18.9)	74 (47.7)
Peri-urban Uganda					
Male	8 (6.1)	101 (77.1)	18 (13.7)	4 (3.1)	131 (45.6)
Female	9 (5.8)	60 (3.8)	51 (32.7)	36 (23.1)	156 (54.4)
Tanzania					
Male	0 (0.0)	7 (24.1)	8 (27.6)	14 (48.3)	29 (17.3)
Female	1 (0.7)	36 (25.2)	50 (36.0)	53 (38.1)	139 (82.7)
Nigeria					
Male	0 (0.0)	13 (31.0)	14 (33.3)	15 (35.7)	42 (35.9)
Female	1 (1.3)	24 (32.0)	29 (38.7)	21 (28.0)	75 (64.1)
South Africa					
Male	0 (0)	28 (20.1)	57 (41.0)	54 (38.8)	139 (29.5)
Female	1 (0.3)	42 (12.7)	88 (26.5)	201 (60.5)	332 (70.5)

### Risk factors for overweight and obesity

Factors found to be associated with overweight and obesity at univariate and multivariate analysis are discussed but the multivariate risk factors by sites, are shown in Table 3.

**Table 3:** Multivariate comparison of factors associated with overweight vs normal weight, obese vs normal weight and overweight/obese vs normal weight, by site

Factor	Rural Uganda	Peri-urban	Nigeria	Tanzania	South Africa
	AOR (95 % CI)	AOR (95 % CI)	AOR (95 % CI)	AOR (95 % CI)	AOR (95 % CI)
Overweight vs Normal weight					
Sex	*p* = 0.35	* *p* < 0.0001	*p* = 0.20	*p* = 0.82	*p* = 0.84
Male	1	1	1	1	1
Female	1.40 (0.66, 2.98)	5.66 (2.69, 11.91)	0.46 (0.14, 1.45)	1.18 (0.35, 3.97)	0.87 (0.46, 1.67)
Age Group, Years	*p* = 0.14	*p* = 0.48	* *p* = 0.002	*p* = 0.91	* *p* < 0.0001
18–34	1	1	1	1	1
35–44	1.18 (0.54, 2.59)	0.98 (0.38, 2.55)	4.16 (1.10, 15.7)	0.58 (0.17, 1.95)	4.49 (1.49, 13.57)
> = 45	0.40 (0.14, 1.11)	1.54 (0.58, 4.08)	13.46	0.94 (0.26, 3.47)	7.90
			(2.19, 82.17)		(2.68, 23.28)
Marital Status	*p* = 0.50	*p* = 0.08	*p* = 0.42	*p* = 0.61	*p* = 0.25
Never married	1	1	1	1	1
Married/living together	1.53	2.47 (1.00, 6.13)	0.49 (0.13, 1.82)	3.18 (0.73, 13.82)	1.36 (0.55, 3.32)
Separated/divorced/widowed	(0.12, 19.17)	2.81 (1.62, 4.88)	Empty	1.68 (0.22, 12.77)	1.78 (0.58, 5.49)
	2.82				
	(0.19, 43.78)				
Obesity vs Normal weight					
Sex	* *p* = 0.002	* *p* < 0.0001	*p* = 0.34	* = 0.92	* *p* = 0.02
Male	1	1	1	1	1
Female	11.22	27.80	0.68	0.81	2.17
	(2.27, 55.40)	(7.13, 108.41)	(0.21, 2.21)	(0.26, 2.51)	(1.19, 4.00)
Age Group, Years	*p* = 0.07	*p* = 1.18	*p* = 0.09	*p* = 0.46	* *p* < 0.0001
18–34	1	1	1	1	1
35–44	0.88 (0.24, 3.21)	1.79 (0.45, 7.10)	0.93 (0.24, 3.65)	0.49 (0.16, 1.52)	3.65 (1.48, 9.00)
> = 45	0.11 (0.01, 1.27)	3.77(0.85, 16.67)	5.19(0.85, 31.88)	0.59 (0.17, 2.06)	5.49(2.27, 13.29)
Marital Status	*p* = 0.31	*p* = 0.07	*p* = 0.21	*p* = 0.41	*p* = 0.82
Never married	1	1	1	1	1
Married/living together	0.33 (0.02, 7.09)	2.22 (0.49, 9.96)	0.40 (0.12, 1.32)	3.36 (0.84, 13.40)	1.26 (0.58, 2.77)
Separated/divorced/widowed	0.19 (0.01, 7.78)	Omitted	Empty	2.30 (0.34, 15.42)	0.96 (0.43, 2.70)
Overweight/Obese vs Normal weight					
Sex	* *p* = 0.04	* *p* < 0.0001	*p* = 0.19	*p* = 0.92	*p* = 0.11
Male	1	1	1	1	1
Female	1.96 (0.96, 3.97)	8.01(4.02, 15.96)	0.52 (0.19, 1.34)	0.98 (0.34, 2.69)	1.57 (0.89, 2.76)
Age Group, Years	*p* = 0.10	*p* = 0.31	* *p* = 0.004	*p* = 0.70	* *p* < 0.0001
18–34	1	1	1	1	1
35–44	1.18 (0.56, 2.49)	1.07 (0.45, 2.52)	2.26 (0.75, 6.79)	0.49 (0.17, 1.44)	3.81 (1.67, 8.70)
> = 45	0.38(0.13, 0.97)	1.79 (0.74, 4.34)	9.11(1.72, 48.16)	0.68 (0.21, 2.21)	6.22 (2.75, 14.07)
Marital Status	*p* = 0.87	*p = 0.01	*p* = 0.18	*p* = 0.43	*p* = 0.53
Never married	1	1	1	1	1
Married/living together	0.90 (0.11, 7.61)	3.93 (1.60, 9.64)	0.43 (0.15, 1.24)	3.48 (1.00, 12.15)	1.27 (0.60, 2.67)
Separated/divorced/widowed	1.33(0.13, 13.77)	3.87(1.10, 13.56)	Empty	2.17 (0.39, 12.09)	1.23 (0.46, 3.26)

* = significant at *p* < 0.05

In rural Uganda (Mbarara), being female was found to be associated with obesity [OR = 9.88; 95 % CI: 2.11, 46.34] and having BMI > =25 kg/m^2^ (overweight and obese) [OR = 2.12; 95 % CI: 1.12, 4.01] at univariate analysis but at multivariate analysis, it was a predictor of obesity after adjusting for age and marital status [(Adjusted Odds Ratio) AOR = 11.22; 95%CI: 2.27, 55.40]. None of the tested explanatory variables were significantly associated with being overweight.

In peri-urban Uganda (Makerere), being female was both a significant risk factor and predictor for the three categories of BMI. This was most with being obese with females having 28 times the odds of being obese [AOR = 27.80; 95 % CI: 7.13, 108.41] compared with males. Other risk factors found to be independently associated with BMI status were age and marital status. Being married/living together [AOR = 3.93; 95 % CI: 1.60, 9.64] and being divorced/separated/widowed [AOR = 3.87; 95 % CI: 1.10, 13.56] each had about 4 times the odds of having BMI > =25 kg/m^2^ compared to those never married.

In Nigeria, it was only age that was found to be significantly associated with and a predictor of overweight and having BMI > =25 kg/m^2^ after adjusting for sex and marital status in logistic regression. Respondents 35–44 years had about 4 [AOR = 4.16; 95 % CI: 1.10, 15.7] and 2 [AOR = 2.26; 95 % CI: 0.75, 6.79] times the odds of being overweight and having BMI > =25 kg/m^2^, respectively compared with those in age category 18–34 years. The risk increased with age; those in age group > =45 years having odds of about 13 [AOR = 13.46; 95 % CI: 2.19, 82.71] and 9 [AOR = 9.11; 95 % CI: 1.72, 48.16] times higher, respectively than those in age category 18–34 years. This trend was also found with being obese but the association was not statistically significant.

In South Africa, age was found to be significantly associated with the three categories of BMI. This remained significant as predictor of the categories after adjusting for sex and marital status. For age group 35–44 years, the odds of being obese, overweight and having BMI > =25 kg/m^2^ were about 4 (AOR = 3.65; 95 % CI: 1.48, 9.00] 5 [AOR = 4.49; 95 % CI: 1.49, 13.57] and 4 [AOR = 3.81; 95 % CI: 1.67, 8.70] times, respectively more than those in age category 18–34 years. The risk increased with age with those in age category > =45 years having about 6 (AOR = 5.49; 95 % CI: 2.27, 13.29], 8 (AOR = 7.90; 95 % CI: 2.68, 23.28] and 6 (AOR = 6.22; 95 % CI: 2.75, 14.07] times the odds of being obese, overweight and having BMI > =25 kg/m^2^, respectively compared with those in age group 18–34 years. In addition, being female, had 2 times the odds of being obese (AOR = 2.17; 95 % CI: 1.19, 4.00] compared with males.

In Tanzania, being female [OR = 2.0; 95 % CI: 1.49, 2.68] compared with males, in age group 35–45 years [OR = 2.07; 95 % CI: 1.42, 3.01] and age group > =45 years [OR = 3.03; 95 % CI: 2.09, 4.39] compared to age group 18–34 years, and married/living together [OR = 2.13; 95 % CI: 1.43, 3.19] and separated/divorced/widowed [OR = 2.81; 95 % CI: 1.62,, 4.88] compared to never married status were found to be significantly associated with being overweight and having BMI > =25 kg/m^2^ at univariate analysis. However none of the explanatory variables tested [sex, age, marital status, and smoking] was significantly associated with BMI status at multivariate analysis.

Table 4 shows the univariate and multivariate risk factors for BMI categories when all sites were combined. In univariate analyses, compared with those of normal weight, sex, age, education, marital status, study site and wealth status were found to be significantly associated with BMI > = 25 kg/m^2^ (overweight and obese). However, in multivariate analysis only being female (AOR) =2.10; 95 % CI: 1.56, 2.84), aged > = 45 years (AOR =1.61; 95 % CI: 1.08, 2.41), married (AOR 1.70 95 % CI: 1.13, 2.56), from South Africa (AOR = 4.97; 95 % CI: 3.18, 7.78), Nigeria (AOR = 2.56; 95 % CI: 1.45, 4.53) and Tanzania (AOR = 2.68; 95 % CI: 1.60, 4.49) were independently associated with having a BMI > = 25 kg/m^2^ (overweight and obese).

**Table 4:** Basic Characteristics of all the participants by BMI status showing Odds Ratio (OR) with 95 % confidence intervals (CI)

Factors	Obesity vs Normal weight		Overweight vs Normal weight		Overweight/obese vs Normal weight	
	Univariate	Multivariate	Univariate	Multivariate	Univariate	Multivariate
	OR (95 % CI)	AOR (95 % CI)	OR (95 % CI)	AOR (95 % CI)	OR (95 % CI)	AOR (95 % CI)
Sex	*p* < 0.0001	*p* < 0.0001	*p* < 0.0001	*P* = 0.005	*p* <0.0001	*P* < 0.0001
Male	1	1	1	1	1	1
Female	3.71 (2.73, 5.04)	2.57 (1.75, 3.78)	2.00 (1.49, 2.68)	1.59 (1.13,2.24)	2.71 (2.11, 3.50)	2.10 (1.56, 2.84)
Age Group, Years	*p* <0.0001	*P* < 0.0001	*p* < 0.0001	*P* = 0.001	*p* <0.0001	*p* < 0.0001
18–34	1	1	1	1	1	1
35–44	2.99 (2.04, 4.38)	1.29 (0.79, 2.11)	2.07 (1.42, 3.01)	1.24 (0.81,1.90)	2.48 (1.80, 3.41)	1.24 (0.85, 1.81)
> = 45	4.97 (3.41, 7.25)	1.82 (1.10, 3.00)	3.03 (2.09, 4.39)	1.67 (1.07, 2.60)	3.89 (2.82, 5.37)	1.61 (1.08, 2.41)
Marital Status	*p* = 0.009	*p* = 0.55	*p* < 0.0001	*p* =0.03	*p* =0.0001	*p* =0.05
- Never Married	1	1	1	1	1	1
- Married/Living together	1.69 (1.17, 2.45)	1.56 (0.95, 2.56)	2.13 (1.43, 3.19)	1.71 (1.06, 2.77)	1.88 (1.36, 2.59)	1.70 (1.13, 2.56)
Separated/divorced/widowed/ other	2.01 (1.18, 3.42)	1.40 (0.68, 2.88)	2.81 (1.62, 4.88)	2.03 (1.04, 3.94)	2.35 (1.48, 3.74)	1.81 (0.99, 3.29)
Country site	*p* < 0.0001	*p* < 0.0001	*p* <0.0001	*P* < 0.0001	*p* <0.0001	*P* < 0.0001
Rural Uganda	1	1	1	1	1	1
Peri-urban Uganda	1.28 (0.68, 2.42)	1.30 (0.64, 2.61)	0.58 (0.37, 0.90)	0.66 (0.40, 1.07)	0.73 (0.49, 1.09)	0.75 (0.48, 1.18)
Nigeria	4.99 (2.46, 10.10)	5.24 (2.41, 11.43)	1.62 (0.93, 2.81)	1.74 (0.94, 3.22)	2.33 (1.42, 3.85)	2.56 (1.45, 4.53)
South Africa	18.94 (10.41, 34.44))	14.10 (7.51,26.49)	2.86 (1.84, 4.45)	2.42 (1.49, 3.94)	6.29 (4.20, 9.42	4.97 (3.18, 7.78)
Tanzania	8.38 (4.32, 16.23)	6.69 (3.32, 13.48)	1.93 (1.15, 3.26)	1.60, (0.90, 2.85)	3.31, (2.06, 5.30)	2.68, (1.60, 4.49)
Educational Status	*p* <0.0001		*p* <0.0001		*p* <0.0001	
Low education	1	–	1	–	1	–
High education	5.98 (4.20, 8.50)	–	1.98 (1.46, 2.67)	–	3.25 (2.49, 4.23)	–
Wealth status	*p* <0.0001		*p* <0.0001		*p* <0.0001	
Low	1	–	1	–	1	–
Middle	2.07 (1.38, 3.09)	–	0.74 (0.52, 1.05)	–	1.15 (0.85, 1.55)	–
High	8.26 (5.43, 12.56)	–	2.42 (1.67, 3.52)	–	4.22 (3.01, 5.90)	–
Current Smoker	*p* = 0.63	–	*p* = 0.92	–	*p* =0.82	–
No	1	–	1	–	1	–
Yes	0.89 (0.56, 1.41)	–	1.02 (0.65, 1.62)	–	0.96 (0.64, 1.42)	–

Results comparing risk factors for obesity vs. normal weight, and for overweight vs. normal weight were very similar to those comparing BMI > =25 kg/m^2^ (overweight and obese) vs. normal weight. However, marital status was not a predictor of obesity.

## Discussion

Overweight and obesity were once associated only with developed countries, however, with urbanization, changes in lifestyle and environment, the prevalence is on the rise in low and middle-income countries, which include those in Africa [21]. Our population-based study involving four sub-Saharan African countries reveals a high prevalence of obesity and overweight, both leading risk factors for many chronic NCDs. This underscores the public health importance of over-nutrition among adults in these sub-Saharan countries, and possibly other countries in the region, as two thirds of all the respondents were overweight and obese (combined). Underweight was barely present, constituting 2 % of all the respondents with a range of less than 1 % in SA to 6 % in peri-urban Uganda. These findings are similar to those of some developed countries [11].

We noted distinct geographical patterns in the prevalence of over-nutrition. The prevalence of overweight and obese combined was 46 %, 48 %, 75 %, 68 % and 85 % in rural Uganda, peri-urban Uganda, Tanzania, Nigeria and South Africa, respectively. Our findings corroborate the high prevalence of over-nutrition reported for these countries in the literature recently and suggest increase in prevalence overtime when compared with the 2013 figures published by Ng et al. The prevalence in each study site is also higher than the average for their region in sub-Saharan Africa (eastern SSA, western SSA and southern SSA) [11]

The variation in BMI status by site supports the urban–rural differences reported in some studies in sub-Saharan Africa [22–24]. We found that participants in our urban sites (South Africa, Nigeria and Tanzania) had higher risk of obesity or overweight compared with the rural and peri-urban Ugandan site. This, corroborates past studies which showed association between urbanization and higher BMI both within and between countries. In Tanzania, a study reported prevalence of obesity to be 13 % and 36 % compared with <10 % and 6 % among urban men and women, respectively [25, 26]. In Uganda, a study reported overweight to be 15.8 % and 23.8 %, and obesity to be 3.9 % and 17.8 %, respectively in rural and urban dwellers [27]. In Nigeria, 40 % and 30 % of females in urban and rural areas were reported to be overweight and obese by Sola et al. [28]. These urban–rural differences have been attributed to nutritional transition whereby there is a dietary shift from traditional diets to processed, energy-rich food, fat, animal-source foods, sugar and sweetened beverages [28]. This dietary shift may be more pronounced among urban compared with rural dwellers because of higher incomes and more availability of processed foods. We also believe that this differential by region could be related to the level of economic development and level of urbanization of the respective countries [29, 30]. Even though we did not test further the association of the wealth index with the BMI categories, due to the fact that 2 of the 5 study sites had nobody in 2 of the wealth categories, there is a suggestion that economic status is a risk factor for overweight and obesity in this study.

In our study, prevalence of over-nutrition and the associated factors also vary across the study sites. South Africa had the highest prevalence of overweight and obesity (combined) at 85 %, and this was higher than the prevalence of 65 % reported in 2012 [23]. The prevalence of obesity (56 %) in SA supports increasing trend when compared with the 23.5 % and 27.2 % reported in 2008 and 2012, respectively in this country [31]. This increasing trend was also found in our other study sites [32]. In Tanzania, prevalence of obesity and overweight was 40 % and 35 %, respectively and this was higher than 32.54 % and 23.44 % reported in 2012, respectively [25]. In Nigeria, the prevalence of overweight and obese in a study in 2011 was 22 % and 4 %, while in 2014 another reported prevalence of 31 % and 17 %, respectively [33] Okafor et al., 2014; these values are much lower than 37 % and 31 % found in our study.

The documented attributable determinants of over-nutrition across African countries including our study sites include female sex, being married, living in urban areas, higher socio-economic category, being African/White, physical inactivity [31, 32, 34] globalization of food production and marketing [34] and increasing age [27]. Furthermore, in Nigeria and South Africa [31, 32], residing in areas of high crime rates have been associated with overweight and obesity because the activities of the criminals prevent the residents from outdoor exercise such as walking due to fear of being harmed. All of these risk factors were corroborated in our study except for residing in high crime rate areas and race which we did not explore. The differences are discussed below.

i) Sex: There was gender differences in BMI status across the study sites. The highest prevalence of obesity was recorded in South African women (61 %) which is about similar to the 69.3 % found by Ng et al. [11]. Within sites, the prevalence of obesity was found to be more among females in SA and the 2 Ugandan sites. This corroborates past studies in these sites where more women were reported to be overweight and obese compared with men. [11. 27]. In addition, our study showed an increasing trend even by sex when compared to a past study in rural and periurban area in Uganda, with overweight among men being 35.5 % vs 12.4 % vs and women 41.9 % vs 23.1 %; but the increase in obesity prevalence was minimal with 2.4 % vs 2.0 % and 18.9 % vs 12.7 % among men and women, respectively [27]. Contrarily, in Nigeria and Tanzania, obesity was more among males while overweight was more among females in our study. This finding, which is similar to the pattern in developed countries, was also reported for Nigeria by Ng et al. in 2013 but not in Tanzania [11]. However, most past studies conducted in Nigeria and Tanzania showed obesity and overweight to be higher among females than males [25, 26, 28, 33]. This deviation in our study despite the fact that majority of our respondents in Nigeria and Tanzania were females, calls for a larger study with more country representative samples.

Sexual variability in obesity and overweight has been adduced to hormonal and genetic differences [35]. However, apart from these, behavioural and cultural factors could be responsible [28]. Being undernourished in childhood and in higher wealth category have been adduced for possible obesity in females [36]. Women engage in less physical activity than men [33] and in some cultures, the practice of female seclusion, as found in Northern Nigeria poses risk for developing obesity [37]. Some authors have reported African cultural factors such as related to body image [38] and men’s preferences for ‘fat’ women who are thought to be more beautiful [30, 33] and the belief that being fat is a sign of affluence [28, 30] to encourage overweight and obesity. This thus calls for interventions that are sensitive to cultural belief systems and values [39] which is especially important in the African context with regards to body image.

Marriage: In our study marriage was found to be associated positively with BMI categories differently though not all were significant. It was only in peri-urban Uganda that we found marital status to be a predictor of BMI status whereby those married/living together and those divorced/separated were 5 and 4 times more likely to be overweight and obese (combined) than never married. In the literature, association between marriage and over-nutrition has been two sided. On one hand marriage was demonstrated to be protective with the argument that marriage promotes better health and increases longevity [40]. On the other hand, entry into marriage has been demonstrated to be a risk factor for obesity. Married couples may no longer pay attention to their weight, they often eat together ordered/fast foods, spend much time watching television together and exercise less [31, 41]. Another risk in females is child bearing. Contrarily, the never married need to keep their weight under check to remain attractive for potential suitors and this may also be true for those divorced especially the females to enhance their prospects in the marriage market [42]. This difference could also be a function of increasing age as those ever married whether still living together or separated/widowed are more likely to be older than the never married. In addition, since this association was found to be significant in only one of the countries studied, cultural differences may be responsibility and needs to be explored.

Age: Age as a predictor of BMI status was demonstrated to be significant only in Nigeria and South Africa. In Nigeria, the risk of being overweight was 4 and 14 times more among those 35–44 years and > =45 years, respectively compared with those younger (18–34 years) and for overweight and obese (combined) it was 2 and 9 times more. However, this finding was for only being overweight and obese (combined) in South Africa with those > =45 years having 2 times the risk of those 35–44 years and 5 times the risk of those younger = <34 years. This finding is similar to that of a study of female teachers in Ghana and another study in rural and peri-urban study in Uganda [27, 43] When the study sites data were pooled together, the risk of overweight and obesity (combined) was also found to increase with age in this study population. This corroborates findings in past studies in both developed and less developed countries [33, 44, 45]. Increased rate of obesity with age has been attributed to hormonal changes and decreased physical activity and metabolism that accompany aging [35].

Occupation, Education and wealth: The education of the respondents in this study is a reflection of the type of profession they engaged in. The respondents were specialized groups comprising teachers in SA and Tanzania, and nurses in Nigeria. However, respondents in the two Ugandan sites were from the general population with a mix of occupation types. The highly selective nature of the respondents was a challenge in the conduct of test of association as Tanzania, Nigeria and South Africa had no one in the lower education category and there was collinearity among the variables. In the same vein, rural Uganda and Tanzania had no participant in the high wealth category. The high prevalence of over-nutrition among those with higher education also reflects the relationship with type of occupation and the wealth index. For this reason we did not dwell on the association of BMI categories with education, occupation type and wealth status. However, over-nutrition has implications in the workplace. It is associated with occupational injury, absenteeism, reduced productivity, weight discrimination, short-term disability, and presentism [43].

The work environment has also been shown to contribute to the obesity epidemic. Such “obesogenic” work environment includes shift work, job stress and long work hours [46]. Health service providers (HSPS) such as doctors, nurses, and pharmacists are one of the most important group of workers facing such “obesogenic” work setting. The work environment of teachers also encourages sedentariness and high prevalence of obesity has been reported among them [43]. However, studies have demonstrated conflicting findings on the association between obesity and health care providers. In developed countries, they have been demonstrated to have lower rate of obesity compared with general population [47] while in developing countries including Nigeria the prevalence among them is similar or in some cases higher than that of the general population [48]. The Nigerian respondents in our study were nurses in Abuja and the prevalence of obesity (31 % %) among them was higher than 17 %% found in the general urban population [33] and lower than the prevalence among nurses in Akwa Ibom (62.6 %) where culturally, females are encouraged to feed well to be “fat” [49]. This high prevalence was also reported among health workers in SA [50].

Wealth or socio-economic status (SES) is a known risk factor for overweight and obesity although it was not tested in multivariate analysis in our study. Albeit, the urban sites recorded more people in the high and middle wealth status compared with the rural and peri-urban sites. Higher SES has predicted higher prevalence of obesity in other studies in Africa [51] contrary to the situation in developed countries, where higher prevalence of obesity is seen among low socioeconomic groups [29, 52]. One of the reasons for high prevalence of overweight and obesity in the low socio-economic class in the developed countries is poverty and access to calorie-dense food which are relatively cheaper than healthy diet. The opposite appears to be true in developing countries where nutrition transition is more prevalent among those of higher socio-economic class since such foods are usually expensive and out of reach of the poorer populace. Unfortunately, in Africa, those in the high socio-economic class who can afford a healthier diet and lifestyle, may not be health cautious. Socio-cultural beliefs that physical inactivity, being ‘fat’ and eating westernised diet are considered as signs of affluence, contribute to inattention to healthy behaviours. Also, many people prefer to be overweight so as not to be perceived as being part of the HIV/AIDS pandemic which has devastated Africa [25].

Our study has some limitations. This was a pilot survey planned to provide data for designing a future large multi-country cohort study, hence, the relatively small sample size in each site. In addition, participants were selected groups which may not be a representative sample of the general population. While we cannot say that estimate of overweight and obesity in this study is generalisable, the findings show that the tsunami of overweight and obesity predicted for developing countries is a reality in our study sites with heterogeneity across the sites [53]. Thus the need for a large prospective study that include more countries in Africa [54]. The choice of professional populations at three sites may have also influenced the relationship between BMI status and wealth status of the respondents. The use of only two household ingredients for determining wealth index in this study is also a limitation and there was nobody in the high wealth status category in 2 (rural Uganda and Tanzania) of the 5 study sites. For education level, three sites did not have anyone with low education reflecting the selectiveness of participants in our study. For this, we deemed it fit not to use these variables in the multivariate analyses. These limitations highlight some of the challenges of multi-country studies.

### Public health implications

We found that the prevalence of overweight and obesity are on the rise in our study sites when compared with past studies and with rural–urban, geographical and gender differences. This has major implications on the healthcare systems in these countries and the region (similar trend in other SSA countries has been reported in the literature) as they will face increasing demand for care of chronic conditions related to obesity and overweight such as diabetes mellitus, osteoarthritis, cancers and cardiovascular diseases. Cardiovascular diseases and diabetes are already among the top leading causes of deaths in these countries [55]. The preponderance of overweight and obesity among the urban dwellers and women points to the need for group specific or targeted interventions to combat the menace. In developing intervention for control of over-nutrition, barriers to lifestyle change at personal, environmental and socio-economic levels should be targeted and stakeholders at different levels should be involved. Policies to regulate dietary habit, provide environments that encourage physical activity behaviours (although not explored in this study), such as creating walk ways, and support health services should be formulated and implemented.

## Conclusion

The prevalence of overweight and obesity was high in the sub-Saharan African populations studied. Although the study was carried out among professionals except in Uganda, the findings reflect the rising incidence of overweight and obesity in developing countries as documented in other recent studies. The observation is that women, older people and those in high socio-economic class were more at risk. Contrarily, in developed countries over-nutrition is seen more among those in low SES and rural populations. Our study also demonstrated that urbanisation (proxy by association with residing in urban areas) is an important determinant of overweight and obesity in our study. As urbanization and the accompanying nutrition transition is bound to continue, the need to stem the rising incidence of obesity becomes pertinent in SSA. This high incidence correlates with the reported escalating NCD prevalence in the region and thus underscores the need for urgent intervention. Raising awareness among those living in urban areas, professionals and women on the role of lifestyle changes in combating obesity and its associated health risks is important. We also recommend that routine measurement of biometrics at every opportunity to identify those at risk of obesity and its complication be instituted in these countries.

## Abbreviations

AIDS: Acquired immunodeficiency syndrome
ANOVA: Analysis of variance
AOR: Adjusted odds ratio
BMI: Body mass index
CVDs: Cardiovascular diseases
DALYs: Disability-adjusted life-years
HIV: Human immunodeficiency virus
HSPH: Harvard T. H. Chan School of Public Health
LMICs: Low and middle-income countries
NCDs: Non-communicable diseases
PaCT: Partnership for cohort research and training
SAS: Statistical analysis system
SES: Socioeconomic status
SSA: Sub-Saharan African
WHO: World Health Organisation.
